# Risk factors for eclampsia in pregnant women with preeclampsia and positive neurosensory signs

**DOI:** 10.4274/tjod.22308

**Published:** 2019-01-09

**Authors:** Houssam Rebahi, Megan Elizabeth Still, Yassine Faouzi, Ahmed Rhassane El Adib

**Affiliations:** 1 Cadi-Ayyad University, Faculty of Medicine and Pharmacy of Marrakech, Department of Anesthesia and Intensive Care Medicine, Marrakech, Morocco; 2 University of Texas Southwestern Medical Center, Clinic of Anesthesiology and Pain Management, Dallas, TX, USA

**Keywords:** Eclampsia, preeclampsia, development, neurosensory signs, risk factors

## Abstract

**Objective:**

In Morocco, eclampsia remains the second major cause of maternal mortality. Conventionally, patients with preeclampsia and neurosensory signs (NSS) (e.g., headaches and hyperreflexia) are considered at high risk of worsening and progressing to eclampsia. However, this specific population is heterogeneous in terms of eclampsia occurrence. We aimed to identify the risk factors for the development of eclampsia in women with preeclampsia presenting with NSS at admission.

**Materials and Methods:**

We performed a single-center, retrospective case-control study of patients with preeclampsia with positive NSS from January 1^st^, 2012 through December 31^st^, 2015, to investigate predictive factors for eclamptic seizures. The case patients were pregnant women with severe preeclampsia who had NSS before developing eclampsia. Control subjects were those with positive NSS without the development of seizures during their hospital stay. One hundred-thirty eight patients with eclampsia and 272 control patients were enrolled.

**Results:**

Univariate analysis revealed that eclampsia was more likely to develop in patients with the following risk factors: maternal age ≤25 years (χ2=9.58, p=0.002), primiparity (χ2=6.38, p=0.011), inadequate prenatal care (χ2=11.62, p=0.001), systolic hypertension ≥160 mmHg (χ2=15.31, p<0.001), diastolic hypertension ≥110 mmHg (χ2=5.7, p=0.017), generalized acute edema (χ2=14.66, p<0.001), hematocrit <35% (χ2=11.16, p=0.001), serum creatinine >100 μmol/L (χ2=13.46, p<0.001), asparate aminotransferase (AST) >70 IU/L (χ2=10.15, p=0.001), and thrombocytopenia (χ2=22.73, p<0.001). Additionally, independent predictive factors for eclampsia in multivariate analysis included inadequate prenatal care [odds ratio (OR), 8.96 [95% confidence interval (CI): 3.9-20.5], p<0.001), systolic blood pressure ≥160 mmHg (OR, 3.130 [95% CI: 1.342-7.305], p=0.008), thrombocytopenia with a platelet count <50.000 (OR, 13.106 [95% CI: 1.344-127.823], p=0.027), AST ≥70 IU (OR, 3.575 [95% CI: 1.313-9.736], p=0.007), and elevated liver enzymes level, and low platelet count (ELLP) syndrome, which is an incomplete variant of HELLP syndrome (H for hemolysis) (OR, 5.83 [95% CI: 2.43- 13.9], p<0.001).

**Conclusion:**

This work highlights two major risk factors in this patient population, inadequate prenatal care and ELLP syndrome, which can help in the early identification of patients at highest risk of developing eclampsia and guide preventive measures.

**PRECIS:** We have attempted, by carrying out a monocentric case-control study, to identify the predicting factors of eclampsia occurrence in a specific population: Preeclamptic women with positive neurosensory symptoms.

## Introduction

Every day, at least 800 women die due to complications of pregnancy and delivery, equaling a death every two minutes and 292.000 deaths per year^(1)^. These complications are particularly overrepresented in developing countries^(2)^. Of these deaths, 12% are related to conditions of hypertension, among which eclampsia is one of the most urgent. Eclampsia is defined by the American College of Obstetricians and Gynecologists as new-onset of grand mal seizures in a woman diagnosed with preeclampsia, and is the second leading cause of maternal mortality in Morocco, after post-partum hemorrhage^(3,4,5)^. The efforts to predict which patients may suffer from preeclampsia and eclampsia have not yet resulted in a meaningful method of prevention, but the identification of certain predictive factors for the development of seizures in patients with preeclampsia can help caregivers optimize their plans and intervention strategies to prevent this serious, deadly pathology. The aim of the study was to identify predictive factors for the development of eclampsia in patients diagnosed with severe preeclampsia with positive neurosensory signs (NSS).

## Materials and Methods

### Study design

This was a retrospective case-control study performed in the Obstetrical Intensive Care Unit (OICU) at the Center for Mothers and Children in the Mohammed VI Hospital Center in Marrakech, Morocco. The inclusion period was from January 1^st^, 2012, through December 31^st^, 2015.

### Patient population

The case group was defined as all patients who experienced preeclampsia with NSS at their admission in the OICU and who developed eclampsia during the inclusion period, and the control group was composed of all patients hospitalized in the same unit for severe preeclampsia with positive NSS. Positive NSS included one or more of the following: 1) persistent headaches resistant to treatment; 2) hyperreflexia, or; 3) visual troubles, including blurry vision, scotoma, floaters, photopsia, or temporal-cortical blindness. All patients included in the study underwent blood tests and, in addition to NSS, exhibited one or more of the following severe features of preeclampsia at admission: 1) Severe hypertension (systolic ≥160 mmHg and/or diastolic ≥110 mmHg); 2) Oliguria (<500 mL/24 hours); 3) serum creatinine concentration >100 µmol/L; 4) thrombocytopenia (platelet count <100.000/mm^3^); 5) impaired liver function as attested by abnormally elevated blood levels of liver enzymes (to twice normal value), or; 6) acute pulmonary edema. Regarding the initial management targeting the stabilization of hospitalized patients, all women received the same local protocol (which is fully concordant with international recommendations), so a magnesium sulfate regimen was given. Nicardipine, a calcium channel blocker, was the first-line antihypertensive medication used and was carefully titrated to gradually lower the mean arterial blood pressure (BP) and mitigate abrupt variations of maternal BP. Moreover, both the preeclamptic parturient and the fetus benefited from close monitoring and surveillance and their statuses were frequently reassessed. Expedited and prompt delivery was considered, regardless of gestational age, in case of any worsening in maternal and/or fetal conditions. For stabilized women with viable fetuses aged less than 34 weeks of gestation, an antenatal schema of corticosteroids (12 mg per day of betamethasone) was administered within 2 days in order to induce fetal lung maturation.

### Exclusion criteria

Patients were excluded if they developed seizures without associated gestational hypertension or had chronic epilepsy, meningitis, toxic or metabolic encephalitis, chronic hypertension, cerebral hemorrhage, or brain tumor.

### Data collection

Data were collected from the archives of patient files in the OICU. Maternal variables gathered included age, past medical history, place of residence, gestational age when admitted to the unit, parity, the time between seizure and delivery, and the evolution of disease while the patient was in the unit. Physical exam variables collected included clinical symptoms at admission, Glasgow Coma score, BP, presence of edema, proteinuria, diuresis, hemoglobin, hematocrit, platelet counts, coagulation panel, liver function, bilirubin, and creatinine.

### Statistical Analysis

Statistical analysis was performed using the SPSS, Version 20 (International Business Machines Corporation) software package. Data related to descriptive analysis were expressed as means, and bivariate analysis was achieved using the chi-square test (eclampsia occurrence was the dependent variable). Variables associated at the p<0.05 level in univariate analysis were entered into a multivariate logistic regression analysis. A final p value <0.05 was considered significant.

## Results

### Patient characteristics

From January 2012 through December 2015, 42.513 patients gave birth in our facility and 429 patients were found to fulfil the study criteria. The number of excluded cases was 19: ten patients due to diabetes, six due to a history of chronic hypertension, and three due to chronic renal insufficiency. The final analysis included 410 patients who met the inclusion criteria, with 138 patients in the study group (eclampsia group), and 272 patients in the control group.

Patient characteristics are detailed in Table 1. Notably, 42.8% of the eclampsia group was under the age of 25 versus only 27.6% of the control group, and the frequency of acute renal insufficiency in the eclampsia group was double that of the control group. Magnesium was used as an anticonvulsant in all cases. Of the 52 patients who had postpartum seizures, 47 (90.4%) were referred for postpartum eclampsia, 29 (61.7%) of whom gave birth at home. These data were unavailable in 16 patients (11.6%). The majority of observed deaths in the two groups results from cerebrovascular accidents (especially hemorrhage, sometimes with atypical locations such as the brainstem), disseminated intravascular coagulopathy, uncontrolled hemorrhagic shock (rupture of liver subscaplular hematoma or placental abruption), and finally multi-organ failure.

### Outcomes

The analysis results are shown in Table 2. After completion of the univariate analysis, 13 independent risk factors for the development of eclampsia were identified as follows: patients aged 25 years old or younger (p=0.002), primiparous women (p=0.011), patients with inadequate prenatal care (p=0.001), patients with systolic BP ≥160 mmHg (p<0.001), diastolic BP ≥110 mmHg (p=0.017), patients with generalized edema (p<0.001), hemoglobin >12 g/dL (p=0.021), hematocrit <35% (p=0.001), thrombocytopenia with platelets <50.000/mm^3^ (p<0.001) or 50.000-99.000/mm^3^ (p<0.001), elevated liver enzymes with asparate aminotransferase (AST) >70 IU/L (p=0.001), creatinine >100 µmol/L (p<0.001), elevated liver enzymes level, and low platelet level (ELLP) (p<0.001), and cesarean birth (p=0.004). In the multivariate analysis, inadequate prenatal care (p<0.001), SBP >160 mmHg (p=0.008), thrombocytopenia with platelets ranging between 50.000 and 99.000/mm^3^ (p<0.001) and <50.000/mm^3^ (p=0.027), AST >70 IU/L (p=0.007), and ELLP syndrome (p<0.001) were independent variables that highly predisposed women with preeclampsia with positive NSS to eclampsia occurrence.

## Discussion

Eclampsia is a serious disease, especially in developing countries where it is a major health issue and one of the leading causes of maternal mortality. The significantly increased incidence of 0.12-3.7% of eclampsia reported in African countriesversus 0.04-0.12% in various European studies may be due to the complex nature of the disease, which is more suitable for a specialized and multi-disciplinary team, often unavailable to most patients in developing countries^(1,6,7,8)^. Multiple risk factors are known for the development of preeclampsia, including primiparity, maternal age over 40 years, chronic hypertension or diabetes, multiple gestations, and a prior history of preeclampsia^(9)^. However, risk factors for the progression to eclampsia are much less clear. The most frequent premonitory signs of eclampsia occurrence are severe hypertension, headaches, ankle clonus, epigastric or right upper quadrant pain, and visual disturbances. However, analysis was not performed on these signs to determine if they constituted statistically significant risk factors for the development of eclampsia in patients already diagnosed with severe preeclampsia, and the degree of hypertension does not appear to consistently predict the risk of eclampsia^(10)^. Therefore, it is imperative to understand other signs and symptoms that may indicate that a patient is at a higher risk for developing eclampsia. In the current study, numerous putative risk factors for eclampsia in patients with severe preeclampsia and positive NSS were identified, particularly a lack of adequate prenatal care, severe hypertension, and signs of HELLP (H for hemolysis) syndrome, which should be addressed to prevent the development of dangerous seizures and reduce both maternal and fetal morbidity and mortality. Most studies to date detail the risk factors for the development of preeclampsia, rather than the development of eclampsia from pre-eclamptic patient cohorts^(11,12)^. In one study, the risk factors for developing eclampsia were similar to those of developing preeclampsia, which included low maternal age, primiparity, obesity, short duration of marriage prior to pregnancy, low level of education, history of preeclampsia, and inadequate prenatal care^(13)^. In our study, all of the above-mentioned risk factors that were analyzed were found to be significant in the univariate analysis. However, only inadequate prenatal care was found to be a significant risk factor upon multivariate analysis. This discrepancy is most likely due to our comparison of patients with severe preeclampsia and patients with eclampsia, rather than analyzing the difference between patients with eclampsia and non-complicated pregnancies. 

It is imperative to be able to identify which patients are at risk of progressing from preeclampsia to eclampsia to initiate preventative measures. In 2011, the Preeclampsia Integrated Estimate of Risk (fullPIERS) model was developed for predicting complications in patients admitted for preeclampsia. This model demonstrated good discriminatory and stratification abilities and has been both internally and externally validated^(14,15,16,17)^. However, the fullPIERS model was designed for use in high-resource settings. In line with this, another model was required for use in low-resource settings that could be easily implemented by mid-level local health care workers because a majority of deaths assigned to disorders of pregnancy occur in low and middle income countries (LMICs), where there is a high incidence of delay in identification of high-risk patients, transportation, and administration of appropriate treatment^(18,19,20)^. Thus, the miniPIERS was developed for use in LMICs. Due to the frequent lack of laboratory data in rural settings, the miniPIERS is based solely on patient demographics, signs, and symptoms. Using a recommended cut-off at 15% for risk of probability of adverse outcome to initiate closer follow-up, and 25% as the cut-off for referral to a tertiary care facility, the miniPIERS has the potential to significantly improve patient care in resource-limited settings^(18)^. However, both the fullPIERS and miniPIERS models were designed to identify patients who are most at risk for developing any complications, not only eclampsia. Therefore, more work must be done to better stratify those at risk and identify which patients have the highest risk of developing eclampsia to begin the proper, specific preventative measures. The goal of early detection of preeclampsia is to be able to introduce effective preventative treatment during pregnancy to avoid eclamptic seizures and their subsequent complications. Indeed, prenatal care has been shown to play an important role in the incidence of eclampsia and other complications of pregnancy,and the lack of proper prenatal care for pregnant women is a major public health concern both in Morocco, as well as other developing countries, where management of high-risk patients is limited, making it challenging to detect early warning signs and act on modifiable risk factors^(21,22,23,24)^. In our work, 89.9% of the patients with eclampsia and 75.7% of the control group were not properly followed prior to admission, conveying a 9 times increased risk of developing eclampsia. This is an important factor to address as there are reliable algorithms published for the calculation of risk of preeclampsia that may be beneficial in the early identification of these patients. As discussed above, one potential intervention for addressing this issue is the implementation of the regular use of the miniPIERS assessment in community healthcare settings. By training community workers to identify the risk factors of the miniPIERS model and use the formula or application to calculate the predicted risk of complications, at-risk patients may be identified earlier and sent to the proper higher-level care facilities. Hypertension is one of the defining characteristics of preeclampsia and severe hypertension with a systolic BP greater than 160 mmHg has been viewed as a warning sign of evolution to eclampsia^(10)^.Based on a recent systematic review of reports of patients with eclampsia, it is unclear whether the degree of hypertension is consistently related to eclampsia^(10)^. However, the results of the present study demonstrate that severe hypertension is a risk factor for the development of seizures in patients with preeclampsia, and therefore should be taken as a sign of condition severity. HELLP syndrome, described for the first time by Weinsteinen in 1982, is the acronym for HELLP. It is generally considered to be a subset of preeclampsia^(25,26)^. Although the laboratory thresholds used to define HELLP syndrome are not unanimous, Sibai proposed the following definition in order to be able to combine and compare the various studies in the literature: hemolysis defined as at least two of the following: 1) lactate dehydrogenase >600 IU/L; 2) presence of schistocytes or; 3) total bilirubinemia >12 mg/L. Additionally, the author defined elevated liver enzymes as AST >70 IU/L and low platelets as a platelet count below 100.000/mm^3(27,28)^. In the present work, bilirubin was the only laboratory value that could be found relating to hemolysis, so the diagnosis of hemolysis, and thus HELLP syndrome, could not be formally made. Therefore, the analysis was performed based on previously described incomplete HELLP syndrome parameters, defined as elevated liver enzymes (EL), ELLP (EL and thrombocytopenia), and isolated low platelet count (LP). On multivariate analysis, AST >70 IU/L, LP (platelet count of 50.000-100.000/mm^3^ and <50.000/mm^3^) and ELLP were found to be independent predictors of eclampsia in this specific patient population. These results are in line with those previously published, which found a statistically significant association between HELLP syndrome and eclampsia, with a 15-25% incidence of HELLP syndrome reported in patients with eclampsia in various studies^(13,16,29)^. The limitations of this study lie chiefly in the methodology: due to the nature of the retrospective chart review, various important data points were missing from our data set, including reliable indicators for hemolysis, the time between the onset of symptoms and admission to our facility, and the severity of the disease upon admission at upstream facilities. Each patient included in the series was given magnesium sulfate, introducing a possible confounding factor as to who may have developed eclampsia in the natural course of the disease. Additionally, some blood tests reported were performed after the onset of eclamptic seizures, introducing possible bias in those data as well. Finally, the inclusion criteria used for this study may have affected the outcome of the analyses because both the case and control patients had severe, late-stage presentation of the disease. This may account for some of the discrepancies between our results and previously published data.

## Conclusion

Eclampsia is a serious public health concern, especially in LMICs where access to proper prenatal care and screening are often not available. It is important to identify risk factors that may increase the probability of patients with severe preeclampsia progressing to eclampsia. We report several major risk factors in the development of eclampsia, including inadequate prenatal care, severe hypertension, and incomplete HELLP syndrome. Thus, it is imperative to address access to care for all pregnant patients, especially in developing countries, treat hypertension early, and to be aware of the importance of incomplete HELLP syndrome to improve maternal and fetal outcomes and work to prevent this devastating condition.

## Figures and Tables

**Table 1:**
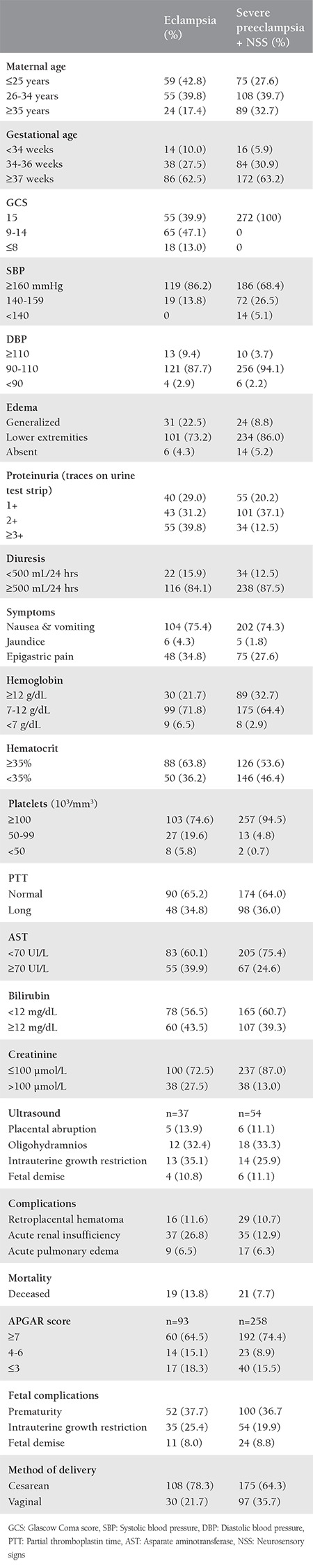
Patient characteristics on admission

**Table 2:**
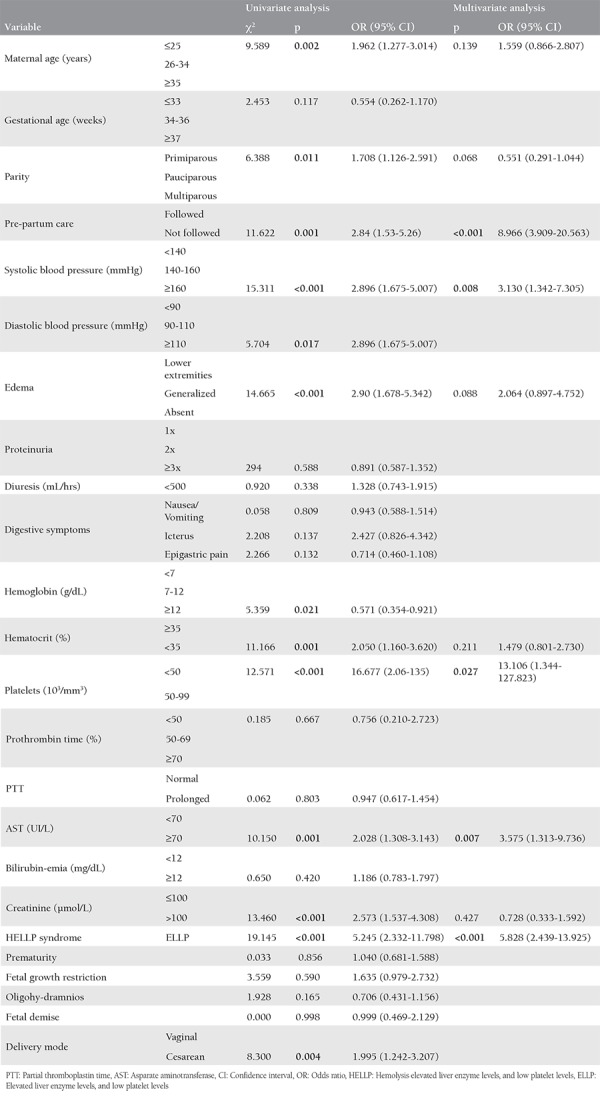
Univariate and multivariate analysis of potential risk factors for the development of eclampsia in patients admitted for severe preeclampsia and positive neurosensory signs
